# Acute treatment with OLX‐07010 reduced tau aggregates and upregulated a marker of autophagy with dose dependence in aged P301L tau JNPL3 mice

**DOI:** 10.1002/alz.092385

**Published:** 2025-01-09

**Authors:** Dhruv R. Patel, Patricia Lopez, Daisy Romero, Eliot J. Davidowitz, James G Moe

**Affiliations:** ^1^ Oligomerix, Inc., Bronx, NY USA; ^2^ Oligomerix, Inc., White Plains, NY USA

## Abstract

**Background:**

OLX‐07010 is an oral small molecule inhibitor of tau self‐association that prevented the accumulation of tau aggregates in the htau mouse model expressing wild type human CNS tau isoforms and in P301L tau JNPL3 mice using chronic treatment by administration in diet (Davidowitz et al., 2020, PMID: 31771053; 2023 PMID:37556474). A therapeutic study of JNPL3 mice with chronic treatment from 7‐12 months of age inhibited the progression of tau aggregation and improved motor coordination. This study evaluated the effect of acute treatment of aged JNPL3 mice using once daily oral dosing of OLX‐07010 for one month.

**Method:**

Serum and brain exposure of OLX‐07010 was determined following a single oral dose via gavage to select the optimal doses for the short‐term treatment study. This study was designed with four groups: Baseline (7 months; n = 15), Vehicle (n = 20) and two treatment groups (20 and 40 mg/kg; n = 20 each). Treatment was administered from nine to ten months‐of‐age once daily by oral gavage. Cortical levels of aggregated tau were determined by two independent methods, SDS‐PAGE to resolve aggregates with immunoblot for detection, and high‐speed centrifugation to pellet aggregates with AlphaLISA for detection. Cortical levels of LC3‐II protein were evaluated using immunoblot. Motor behavior was evaluated from 7‐10 months of age using Open Field Test and Rotarod assays. The primary endpoint was reduction of aggregated tau with statistical significance; secondary endpoints included dose‐dependent reduction of tau aggregation and rescue of motor impairment.

**Result:**

This work characterized the exposure of orally dosed OLX‐07010 in the blood and brain and demonstrated a dose‐dependent reduction of tau aggregates to baseline levels in conjunction with a dose‐dependent increase of LC3‐II, a marker for autophagosome levels, in the cortex and amelioration of the motor impairment phenotype in aged JNPL3 mice, all with statistical significance.

**Conclusion:**

Four weeks of daily oral dosing was sufficient to demonstrate a dose dependent therapeutic effect on tau pathology and motor function supporting clinical development of OLX‐07010. This molecule, selected for inhibiting tau self‐association in vitro, may also improve the clearance of protein aggregates in vivo, further supporting its therapeutic potential.

